# Consumer-Driven Improvement of Maize Bread Formulations with Legume Fortification

**DOI:** 10.3390/foods8070235

**Published:** 2019-06-29

**Authors:** Luís M. Cunha, Susana C. Fonseca, Rui C. Lima, José Loureiro, Alexandra S. Pinto, M. Carlota Vaz Patto, Carla Brites

**Affiliations:** 1 GreenUPorto & LAQV/REQUIMTE, DGAOT, Faculdade de Ciências da Universidade do Porto, Campus de Vairão, Rua da Agrária 747, 4485-646 Vila do Conde, Portugal; 2 Sense Test, Lda., R. Zeferino Costa 341, 4400-345 Vila Nova de Gaia, Portugal; 3 Patrimvs Indústria, SA, Zona Industrial da Abrunheira, 33/34 Apt 184, Vila Chã, 6270-187 Guarda, Portugal; 4 INIAV-Instituto Nacional de Investigação Agrária e Veterinária, 2780-157 Oeiras, Portugal; 5 ITQB NOVA-Instituto de Tecnologia Química e Biológica António Xavier, Universidade Nova de Lisboa, Av República, Apartado 127, 2781-901 Oeiras, Portugal

**Keywords:** maize bread, legume fortification, pea, chickpea, faba bean, lentil, protein content

## Abstract

The fortification of maize bread with legume flour was explored in order to increase the protein content of the traditional Portuguese bread ‘*broa*’, comprised of more than 50% maize flour. The optimization of legume incorporation (pea, chickpea, faba bean, lentil), considering the influence of different maize flours (traditional-white, traditional-yellow, hybrid-white, hybrid-yellow), on consumer liking and sensory profiling of ‘*broa*’ was studied. A panel of 60 naïve tasters evaluated twenty different breads, divided in four sets for each legume flour fortification, each set including four breads with varying maize flour and a control (no legume). Tasters evaluated overall liking and the sensory profile through a check-all-that-apply ballot. Crude protein and water content were also analyzed. There were no significant differences in overall liking between the different types of legumes and maize. The incorporation of chickpea flour yields a sensory profile that most closely resembles the control. The protein content increased, on average, 21% in ‘*broa*’, with legume flours having the highest value obtained with faba bean incorporation (29% increase). Thus, incorporation of legume flours appears to be an interesting strategy to increase bread protein content, with no significant impact on consumer liking and the ‘*broa*’ bread sensory profile.

## 1. Introduction

Bread, a cereal-based product, is an important part of the human diet but rich in easily digested carbohydrates that are associated with a high glycemic index food consumption, which is a health concern for present consumers. Wheat is the most frequently used cereal for bread making in many parts of the world, but other common ingredients of bread are maize and rye [1]. An interesting alternative to wheat bread, with a lower glycemic index, is a maize-based bread named ‘*broa*’ [2]. 

‘*Broa*’ is a Portuguese bread comprised of more than 50% maize flour, mixed with either wheat and/or rye flours, and highly consumed in the northern and central regions of Portugal [3]. Several types of ‘*broa*’ can be produced, depending on the type of maize variety and blending of the flours used, with regional maize landraces (normally open pollinated varieties, OPV) being considered more suitable than hybrid varieties for bread production [4,5]. Maize flour parameters related to the consumer perceived quality of Portuguese ‘*broa*’ bread, based on eleven regional OPV maize landraces, were evaluated [6]. The study revealed similar hedonic assessments (appearance, odor, texture, flavor, color, global appreciation, and cohesiveness) of ‘*broa*’ bread among specialty landraces of maize flours and the lowest scores for ‘*broa*’ bread from commercial (hybrid variety) maize flour. In that study, commercial flour presented the highest mean diameter and a larger flour particle distribution range of all the tested maize varieties. 

The traditional bread making process used to prepare ‘*broa*’ consists of mixing maize flour (sieved whole meal flour), wheat and/or rye flour, hot water, yeast, salt, and leavened dough from a previous bread (acting as the sourdough) [3]. After mixing and resting, the dough is baked in a wood-fired oven. This empirical process leads to an ethnic product highly appreciated for its distinctive sensory characteristics (unique flavor and texture) and provides an interesting source of nutritional value [2,3,7]. The microbiological profile of flours used to manufacture ‘*broa*’ bread and the microbial phenomena of dough fermentation and storage for ‘*broa*’ bread was studied in [7,8,9].

A gluten-free ‘*broa*’ bread, with modification of the traditional composite maize/rye wheat flour, was tested and considered satisfactory for its sensory quality and bread making technology ability [10]. Maize dough has no gluten proteins, which enables it to hold the gas produced during fermentation in a viscoelastic network, leading to a compact bread with crumb-like texture and low specific volume [5].

Legumes are generally rich in protein and fiber and low in fat, and are considered to have a high nutritional value and key role in preventing metabolic diseases, such as diabetes mellitus and coronary heart diseases [11,12]. Thus, legumes can contribute significantly to the protein fortification of cereal-based products to align them with the high vegetal protein diet trend [13]. Legume-enriched bread may be amenable with claims such as ‘source of’, ‘high’ or ‘increased of’ vegetal protein according with Reg EC 1924/2006 [14]. Despite nutritional enrichment, the organoleptic quality of legume-fortified cereal foods was significantly different and tends to decline [15,16,17] when compared with formulations based exclusively on cereals. Thus, the assessment of consumer acceptability is essential to promote the incorporation of legume flours in cereal-based products. The pre-treatment of grain legumes (roasting, cooking, or fermentation) influences the composition and protein properties of grain legumes and, consequently, the characteristics of dough and bread fortified with legume flours [18].

The objectives of this work were to select the optimal maize bread formulation with legume fortification (part of maize flour replaced by legume flour) accessed through overall consumer liking and a check-all-that-apply (CATA) profiling evaluation, in order to obtain bread claiming to have a high protein content that is also nutritiously enriched and well accepted by consumers.

## 2. Materials and Methods 

### 2.1. Experimental Procedure

The base formulation of the ‘*broa*’ bread included 700 g·kg^−1^ maize, 200 g·kg^−1^ rye, 100 g·kg^−1^ wheat flour, 28 g·kg^−1^ sugar (wt/wt flour basis), 17.6 g·kg^−1^ salt (wt/wt flour basis), 10 g·kg^−1^ dry yeast (wt/wt flour basis), 100 g·kg^−1^ sourdough (wt/wt flour basis), and 100% (vol/wt flour basis) water, as described previously by Brites et al. [2].

Twenty breads were produced following a 4-block design combining four legume flours: chickpea—CH (*Cicer arietinum*), faba bean—FB (*Vicia faba*), lentil—LC (*Lens culinaris*), pea—PS (*Pisum sativum*), and control—C (without legume flour) with four different maize flours: hybrid white—IW, hybrid yellow—IY, regional white—RW, regional yellow—RY. The regional whole maize flours were obtained after milling the grain in an artisanal water mill with millstones (Moinhos do Inferno, Viseu), and the hybrid flours correspond to commercial maize flour (Nacional type 175). Each of the four blocks corresponds to the replacement of 10% of the maize flour by one of the legume flours (CH, FB, LC, PS) and a control (C) sample with no replacement.

**Figure 1:**
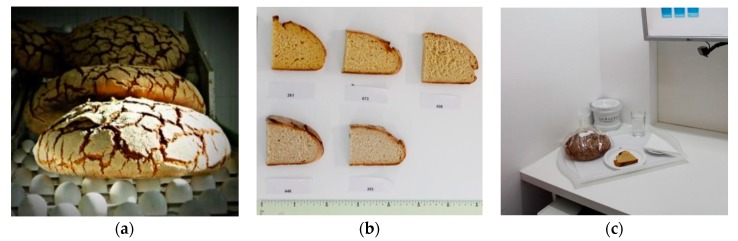
(**a**) ‘*Broa’* bread after baking; (**b**) Slices of ‘*broa’* bread samples for sensory evaluation; (**c**) Individual booth with sample for sensory evaluation.

The ‘*broa*’ bread making process, performed at Patrimvs Indústria, a bakery industry in Portugal, was previously described in [2] and consisted of mixing the maize flour with 80% of the boiling salted water and kneading for 5 min (Ferneto AEF035). The dough was allowed to rest and cool to 27 °C, and the remaining ingredients (sugar, salt, dry yeast, sourdough), including 20% of the water, were added. The dough was again kneaded for 8 min and left to rest for bulk fermentation at 25 °C for 90 min. After fermentation, the dough was manually molded into 400 g balls and baked in an oven (Matador, Werner & Pfleiderer Lebensmitteltechnik GmbH, Dinkelsbühl, Germany) at 270 °C for 40 min (Figure 1a). 

Maize breads produced by Patrimvs Indústria were packed and dispatched the day before each of the sensory evaluation sessions, according to the different legume flour blocks.

### 2.2. Sensory Analysis

Sixty naïve tasters who consumed bread regularly were recruited for a descriptive profiling test from Sense Test’s (an independent Sensory Analysis Laboratory in Portugal) consumer database. A sociodemographic characterization of consumers was performed. The company ensures data protection and confidentiality through the authorization 2063/2009 awarded by the National Data Protection Commission and an accomplished internal code of conduct.

Sensory evaluation was carried out at Sense Test in a special room equipped with individual booths in accordance with ISO standard 8589:2007 [19], with personnel and panel leader following ISO standards 13300-1:2006 [20] and 13300-2:2006 [21].

Four sessions were set up and, for each session, five different samples of maize bread were produced: (i) white regional maize with legume flour; (ii) white hybrid maize with legume flour; (iii) yellow regional maize with legume flour; (iv) yellow hybrid maize with legume flour; and a control sample made with yellow hybrid maize with no legume flour (Figure 1b). ‘*Broa*’ breads were cut halfway and 2 cm-thick slices were cut from the central portion. A serving of one slice (≈100 g) was presented to each taster on white disposable plastic plates, identified by a three-digit random number, at the individual booths under normal white lighting (Figure 1c). Panelists were provided with a porcelain spittoon, a glass of bottled natural water, and unsalted crackers. All panelists were instructed to chew a piece of cracker and to rinse their mouth with water before testing each sample. Panelists were free to swallow or spit both samples and crackers.

Within a session, each participant received all the samples following a monadic sequential (one at a time) presentation, with a balanced sample serving order to compensate for possible carryover effects [22]. Overall liking was evaluated using the classical 9-point hedonic scale, going from 1—“dislike extremely” to 9—“like extremely” [23]. For each sample, overall liking scoring was immediately followed by the evaluation of the sensory profile, through a check-all-that-apply (CATA) methodology to reduce bias [24,25].

Participants were invited to profile each sample over a CATA ballot, structured according to 6 sensory dimensions (Whole bread appearance (WBA), Slice appearance (SA), Texture at touch (TT), Mouth texture (MT), Aroma (A), and Flavor (F)). Table 1 presents the total 51 attributes, according to the respective sensory dimension. The CATA ballot was generated after discussion between the authors based on previous research [26]. This list was presented in two different orders to the panelists, following a direct and an inverse alphabetic order within each dimension [25]. The CATA ballot, “Please select out of the following list of terms those that characterize the tasted sample”, was answered as a “yes/no” response scale, indicating if they recognized the presence or absence of such attributes. This option increased the focus of respondents on each attribute [27].

### 2.3. Protein and Water Content Determination

The bread samples were prepared according to the AACC 62-05.01 method [28], water content by the ISO 712:2009 [29], and protein content by the combustion method of ISO 16634-2/TS:2016 [30], calculated by multiplying nitrogen concentration by a conversion factor of 5.7.

### 2.4. Statistical Analysis

Data analyses were performed using the XL-STAT 2019^®^ system software (Addinsoft, New York, NY, USA). To synthesize the results of the overall liking test, descriptive statistics with mean and standard error (SE) for each session, corresponding to the different legume flour breads and the control sample, were computed. A two-way (type of maize and type of legume) ANOVA with blocks (tasters), and the Fisher-LSD test for multiple comparison (differences in the liking of each of the enriched breads), was applied at a 95% confidence level. A two-way (type of maize and type of legume) ANOVA, and Fisher-LSD test for multiple comparison, was applied to evaluate differences between both the protein and water content of the different ‘*broa*’ breads (with interaction as the error) at a 95% confidence level.

For CATA evaluations, the Cochran test was applied to identify which descriptive attributes were discriminating among samples [31]. Subsequently, the frequency of use of each attribute was determined, calculating the number of panelists who have used each attribute to describe the samples. Over this frequency matrix, a correspondence analysis (CA) was applied. Such analysis provides a sensory map of the bread samples, allowing the perception of the similarities and differences between samples and their sensory characteristics [32,33,34]. Multidimensional alignment (MDA) was also performed to determine the correlation between the descriptive attributes and the bread samples in the full-dimensional space of the CA, providing complete information about the relationship between products and attributes [35].

## 3. Results and Discussion

Generally, the overall liking for all types of breads presented high values of acceptability, around 7—“like very much” (Table 2). Breads with legume incorporation tended to have a somewhat lower acceptability than the control samples (Table 2). Significant differences in the overall liking of the control samples used in each session were identified. This was probably due to industrial variability, as batches were produced in different time periods. To overcome this effect, differences of overall liking of each of the legume-incorporated breads, and the respective control bread, were calculated. From the two-way ANOVA model with panelists as blocks, no significant effects were found for the type of legume, type of maize as well, as for the interaction of both factors (*p* > 0.05). Despite no significant effect, the bread incorporating chickpea flour with hybrid white maize was the only one presenting an average overall liking (6.97 ± 0.16) above the corresponding control sample (6.77 ± 0.17) (Table 2).

Results from the CATA profiling by Cochran test yielded both discriminating and non-discriminating attributes. The non-discriminating attributes identified across all blocks are presented in Table 3. It is important to highlight that the non-discriminating terms associated with the legume incorporation, such as the chickpea, lentil, and pea aroma and the chickpea and faba bean flavor, indicated that the presence of the respective legume flours was not noticeable to consumers.

**Figure 2:**
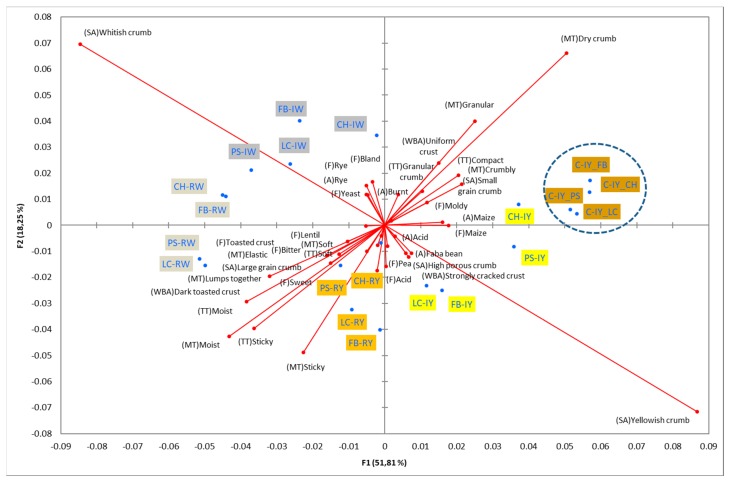
Sensory profiling for bread fortification with legume flours, produced combining 4 legume flours (CH—chickpea, FB—faba bean, LC—lentil, and PS—pea) and a C—control (without legume flour) with 4 maize flours (IW—hybrid white, IY—hybrid yellow, RW—regional white, RY—regional yellow).

Figure 2 shows the configurations of the samples and discriminating descriptive terms in the first and second dimensions of the CA analysis, applied to the CATA data of bread samples. This configuration explained 78.06% of the total variance of the experimental data. From the analysis of Figure 2, it is possible to observe that the samples were grouped according to flour maize type, and that the hybrid white (IW) and regional white (RW) maize were strongly associated with (SA) Whitish crumb, in contrast to hybrid yellow (IY) and regional yellow (RY) maize breads that were intensely associated with (SA) Yellowish crumb, as expected. Data yielded a very high consistency on the profiling of the control samples across all 4 blocks (circle lined in Figure 2), correlated with (SA) Yellowish crumb, (MT) Dry crumb, (TT) Compact, (MT) Crumbly, (A) Maize, and (F) Maize. The incorporation of chickpea flour yielded the sensory profile that most closely resembled the control samples. The regional maize flours were closer associated with sticky and moist descriptors, related with bread crumb cohesiveness (sticky texture at touch and in mouth), and apparent humidity (perceived moisture at touch and in mouth).

Table 4 shows the results from the cosines of the angles of ‘*broa*’ bread samples, with the significant descriptive attributes, resulting from MDA analysis. Angles below 45° indicate a significant positive correlation between the projection of the sample and the projection of the attribute into the CA space, while angles above 135° indicate a significant negative correlation between the projection of the sample and the projection of the attribute [35]. From this analysis, it was possible to depict, in a more detailed way, the differences in bread samples and their relationship with the descriptive terms, since MDA is a statistical procedure that takes into account all the dimensions. The association between (SA) Whitish crumb and (SA) Yellowish crumb, according to the white and yellow maize respectively, was again evident in Table 4. The positive association with the acid flavor for faba bean, lentil, and pea was highlighted in this analysis.

Table 5 presents the protein and water content of the ‘*broa*’ bread produced, combining the 4 legume flours and the control (without legume flour) with the 4 maize flours. Legume fortification significantly increased the ‘*broa*’ bread protein content, as expected. The mean protein content of the bread without legume incorporation was 56.2 (±1.3) g·kg^−1^ and increased, on average, by 21% in ‘*broa*’ with the incorporation of legume flours. The highest increase in protein content was obtained with faba bean (29%) incorporation rising to 72.3 (±2.4) g·kg^−1^, compared to the control without legume incorporation. In terms of protein content per dry weight, the differences were even higher between legume fortification and the control bread. The faba bean incorporation increased the protein content to 32% (118.3 (±1.8) g·kg^−1^ dry basis), followed by lentil incorporation of 22% (108.8 (±0.8) g·kg^−1^ dry basis), and chickpea and pea incorporation (17% and 16%, respectively). The lowest water content was obtained from the bread with pea incorporation (362.5 (±5.6) g·kg^−1^) and the highest from faba bean incorporation (389.5 (±12.4) g·kg^−1^). A significantly lower water content was obtained from the hybrid varieties of bread in comparison with the regional varieties of bread.

## 4. Conclusions

Incorporation of legume flours appears to be an interesting strategy to increase bread protein content without decreasing consumer liking. However, further research should also consider the impact of legume incorporation on the glycemic index of maize bread. Regarding the sensory profile method, one can observe that the CATA was an appropriate method to describe the maize bread formulations with legume fortification. The breads were produced in the bakery chain of Patrimvs S.A, with the intended positive effect of studying a real-life market production situation, but the industrial scale imposed limitations, such as the restricted choice of commercially available legumes flours. Major changes in the ‘*broa*’ sensory profile appear related to apparent humidity (perceived moisture at touch and in mouth) and bread crumb cohesiveness (sticky texture at touch and in mouth). Incorporation of chickpea flour lead to liking scores closer to the control formulation. The incorporation of chickpea flour yielded the sensory profile that most closely resembled the control. Faba flour incorporation lead to ‘*broa*’ breads with the highest protein content. 

These results can be seen as an opportunity for the bakery industry to develop new products that will respond to the growing consumer demand for high-protein food.

## Figures and Tables

**Table 1:** Dimensions and descriptive attributes used in check-all-that-apply (CATA) ballot.

Dimension	Number of Attributes	Descriptive Attributes
Whole bread appearance (WBA)	3	Dark toasted crust, Uniform crust, Strongly cracked crust
Slice appearance (SA)	6	Whitish crumb, Yellowish crumb, High porous crumb, Large grain crumb, Small grain crumb, Homogeneous porosity crumb
Texture at touch (TT)	5	Moist, Soft, Granular crumb, Compact, Sticky
Mouth texture (MT)	11	Crumbly, Hard crust, Crunchy crust, Elastic crust, Dry crumb, Sticky, Moist, Elastic, Granular, Soft, Lumps together
Aroma (A)	11	Intense, Acid, Maize, Rye, Pea, Faba bean, Chickpea, Lentil, Yeast, Burnt, Moldy
Flavour (F)	15	Toasted crust, Intense, Sweet, Acid, Bitter, Salty, Bland, Pea, Faba bean, Chickpea, Lentil, Maize, Rye, Moldy, Yeast

**Table 2:** Mean values ± standard error (SE) of overall liking of control breads (C-IY) and breads combining four legume flours (CH—chickpea, FB—faba bean, LC—lentil, and PS—pea) with 4 maize flours (IW—hybrid white, IY—hybrid yellow; RW—regional white, RY—regional yellow), using a 9-point scale, going from 1—“dislike extremely” to 9—“like extremely”.

Session	Sample	*n*	Overall Liking	
1	C-IY	60	6.77 (±0.17) ^a^	
	CH-IW	60	**6.97 (±0.16)** ^a^	6.63 (±0.09) (*n* = 240)
	CH-IY	60	6.53 (±0.18) ^a^	
	CH-RW	60	6.50 (±0.20) ^a^	
	CH-RY	60	6.52 (±0.16) ^a^	
2	C-IY	60	7.22 (±0.15) ^a^	
	FB-IW	60	6.95 (±0.12) ^a^	6.93 (±0.08) (*n* = 240)
	FB-IY	60	7.20 (±0.14) ^a^	
	FB-RW	60	6.90 (±0.16) ^a^	
	FB-RY	60	6.68 (±0.17) ^a^	
3	C-IY	60	7.22 (±0.18) ^a^	
	LC-IW	60	6.98 (±0.17) ^a^	6.95 (±0.08) (*n* = 240)
	LC-IY	60	7.00 (±0.14) ^a^	
	LC-RW	60	6.97 (±0.17) ^a^	
	LC-RY	60	6.83 (±0.17) ^a^	
4	C-IY	60	7.08 (±0.15) ^a^	
	PS-IW	60	6.88 (±0.15) ^a^	6.89 (±0.07) (*n* = 240)
	PS-IY	60	6.92 (±0.14) ^a^	
	PS-RW	60	7.00 (±0.15) ^a^	
	PS-RY	60	6.75 (±0.16) ^a^	

^a^ within each session, the same letter indicates no significant differences between ‘*broas*’, according to the Fisher-LSD test (*p* > 0.05).

**Table 3:** Dimensions and descriptive attributes of non-discriminating attributes across all blocks.

Dimension	Descriptive Attributes
Slice appearance (SA)	Homogeneous porosity crumb
Mouth texture (MT)	Crunchy crust, Elastic crust, Hard crust
Aroma (A)	Chickpea, Intense, Lentil, Moldy, Pea, Yeast
Flavor (F)	Chickpea, Faba bean, Salty, Intense

**Table 4:** Multidimensional alignment (MDA) for bread fortification with legume flours, produced combining 4 legume flours (CH—chickpea, FB—faba bean, LC—lentil, and PS—pea) with 4 maize flours (IW—hybrid white, IY—hybrid yellow, RW—regional white, and RY—regional yellow). Significant correlations between samples and attributes are depicted with the bold bars.

Legume	Type of Maize			
	IW	IY	RW	RY
CH	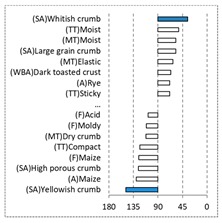	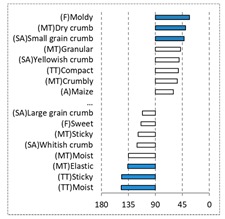	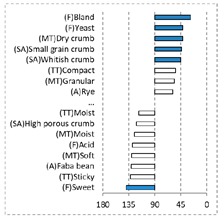	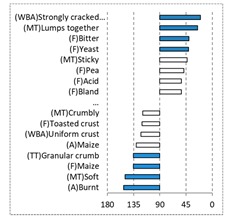
FB	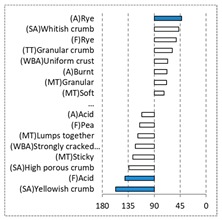	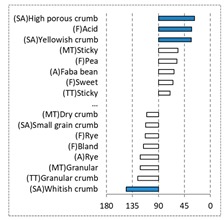	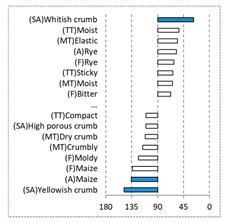	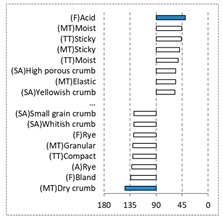
LC	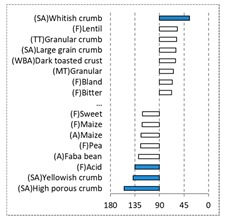	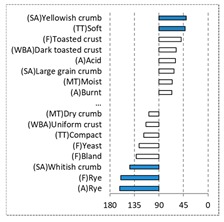	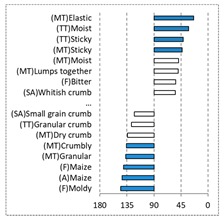	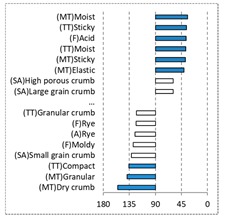
PS	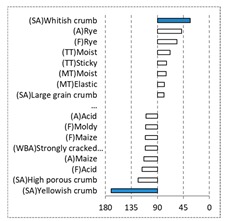	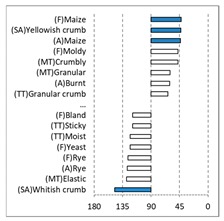	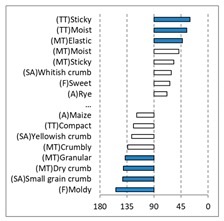	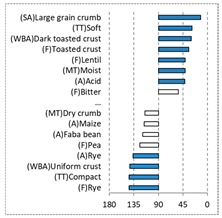

**Table 5:** Mean values ± SE of protein and water content of the ‘*broa*’ breads produced combining 4 legume flours (CH—chickpea, FB—faba bean, LC—lentil, and PS—pea) and C—control (without legume flour) with 4 maize flours (IW—hybrid white, IY—hybrid yellow, RW—regional white, RY—regional yellow).

‘*Broa*’ Sample	Protein Content (g·kg^−1^)	Protein Content in a Dry Basis (g·kg^−1^ Dry Basis)	Water Content (g·kg^−1^)
C	56.2 (±1.3) ^a^	89.5 (±1.0) ^a^	372.0 (±10.7) ^a,b^
CH	65.6 (±1.7) ^b^	105.0 (±1.4) ^b^	375.3 (±10.5) ^a,b^
FB	72.3 (±2.4) ^c^	118.3 (±1.8) ^d^	389.5 (±12.4) ^b^
LC	67.7 (±1.4) ^b^	108.8 (±0.8) ^c^	377.3 (±12.1) ^a,b^
PS	66.3 (±0.4) ^b^	104.0 (±1.1) ^b^	362.5 (±5.6) ^a^
IW	66.1 (±1.9) ^a,b^	104.0 (±4.1) ^a^	363.4 (±11.4) ^a^
IY	68.0 (±3.7) ^b^	106.0 (±5.3) ^a,b^	358.8 (±3.6) ^a^
RW	65.3 (±2.4) ^a,b^	107.0 (±4.5) ^b^	388.8 (±6.8) ^b^
RY	63.0 (±3.0) ^a^	103.4 (±4.9) ^a^	390.2 (±4.0) ^b^

^a,b,c,d^ Similar letters indicate homogeneous groups according to the Fisher-LSD test (*p* > 0.05).
